# Supplementary material for: Predictive Value of the Advanced Lipoprotein Profile and Glycated Proteins on Diabetic Retinopathy
Source: Nutrients. 2022 Sep 22;14(19):3932. doi: 10.3390/nu14193932 (PMC9572733; doi:10.3390/nu14193932)
Supplement: Supplementary file 1 [file nutrients-14-03932-s001.zip › nutrients-1894334-supplementary.pdf]

Supplementary Material

Table S1. Clinical characteristics according to diabetes type and presence or degree of diabetic retinopathy.

Characteristics	T1D				T2D			
	**No DR** *n* = 181	**Mild** *n* = 80	**Advanced** *n* = 48	***p*-value**	**No DR** *n* = 139	**Mild** *n* = 50	**Advanced** *n* = 75	***p*-value**
Age (years)	44.0 (10.9)	47.8 (12.5)	50.8 (11.3)	<0.001	57.4 (10.0)	58.4 (8.80)	60.1 (8.43)	0.126
Sex (women)	95 (52.5%)	41 (51.2%)	27 (56.2%)	0.855	66 (47.5%)	20 (40.0%)	40 (53.3%)	0.342
BMI (kg/m^2)	25.4 (3.86)	26.6 (3.93)	27.2 (4.78)	0.008	31.3 (5.25)	32.0 (5.60)	31.7 (5.74)	0.761
Waist (cm)	87.8 (11.7)	91.7 (11.7)	92.1 (15.0)	0.015	104 (12.1)	106 (9.01)	107 (11.6)	0.207
sBP (mmHg)	125 (17.5)	131 (15.4)	135 (23.0)	0.002	134 (15.7)	137 (19.6)	148 (20.2)	<0.001
dBP (mmHg)	74.6 (10.1)	74.4 (9.15)	72.8 (11.5)	0.544	76.4 (10.2)	75.7 (11.2)	77.9 (10.6)	0.440
Hypertension (yes)	35 (19.3%)	26 (32.5%)	27 (56.2%)	<0.001	68 (48.9%)	32 (64.0%)	49 (65.3%)	0.034
Dyslipidemia (yes)	68 (37.6%)	38 (47.5%)	28 (58.3%)	0.025	61 (43.9%)	28 (56.0%)	35 (46.7%)	0.338
Smoking:				0.855				0.727
Yes	47 (26.0%)	20 (25.0%)	11 (22.9%)		29 (20.9%)	12 (24.0%)	14 (18.7%)	
Former smoker	43 (23.8%)	24 (30.0%)	13 (27.1%)		50 (36.0%)	15 (30.0%)	22 (29.3%)	
Diabetes duration (years)	17.9 (9.72)	25.4 (10.0)	30.2 (9.23)	<0.001	6.88 (5.48)	12.7 (10.1)	14.2 (9.13)	<0.001
Glucose (mg/dL)	163 (71.6)	174 (77.7)	163 (80.2)	0.512	148 (49.2)	153 (51.8)	174 (60.9)	0.003
Creatinine (mg/dL)	0.77 (0.16)	0.76 (0.14)	0.79 (0.15)	0.546	0.81 (0.17)	0.79 (0.15)	0.82 (0.18)	0.522
eGFR (mL/min/1.73 m^2)	103 (13.9)	102 (13.5)	95.9 (14.0)	0.004	92.0 (14.5)	94.5 (11.9)	87.7 (15.6)	0.025
Triglycerides (mg/dL)	72.6 (29.5)	82.7 (44.5)	84.2 (54.8)	0.057	136 (68.9)	151 (77.6)	121 (63.6)	0.065
Total C (mg/dL)	179 (28.1)	180 (35.1)	182 (33.5)	0.879	186 (36.4)	186 (38.7)	185 (35.4)	0.963
HDL-C (mg/dL)	64.3 (14.4)	62.0 (18.6)	63.8 (14.6)	0.540	48.1 (10.6)	49.5 (15.1)	54.0 (15.6)	0.007
LDL-C(mg/dL)	101 (23.1)	102 (27.6)	102 (29.3)	0.915	112 (30.6)	106 (31.5)	107 (29.5)	0.434
HbA1c (%)	7.48 (0.94)	7.78 (1.11)	8.05 (1.04)	0.001	7.29 (1.16)	8.19 (1.43)	8.47 (1.48)	<0.001

HbA1c (mmol/mol)	58.3 (10.3)	61.6 (12.1)	64.4 (11.4)	0.001	56.1 (12.7)	66.1 (15.6)	69.1 (16.2)	<0.001
Plaque:				<0.001				<0.001
Multiple plaques	21 (11.6%)	13 (16.2%)	20 (41.7%)		28 (20.1%)	17 (34.0%)	37 (49.3%)	
No	134 (74.0%)	50 (62.5%)	19 (39.6%)		69 (49.6%)	21 (42.0%)	21 (28.0%)	
One plaque	26 (14.4%)	17 (21.2%)	9 (18.8%)		42 (30.2%)	12 (24.0%)	17 (22.7%)	
FLI	24.3 (22.1)	31.0 (24.3)	36.1 (28.8)	0.004	66.8 (22.7)	72.1 (20.7)	67.7 (24.3)	0.364
Microalbuminuria (mg/L)	9.26 (24.4)	10.5 (22.9)	19.5 (27.9)	0.039	11.1 (14.7)	25.4 (35.1)	33.2 (38.4)	<0.001
ACR (mg/g)	4.48 (11.0)	4.86 (16.8)	8.61 (23.2)	0.234	9.93 (18.6)	20.5 (30.4)	35.9 (43.9)	<0.001

Data are shown as n (%) for categorical variables and mean (SD) for continuous variables. ACR, albumin-to-creatinine ratio; BMI, body mass index; C, cholesterol; dBP, diastolic blood pressure; DM, Diabetes Mellitus; eGFR, estimated glomerular filtration rate calculated according to Chronic Kidney Disease Epidemiology Collaboration (CKD-EPI) equation; FLI, fatty liver index; HbA1c, glycated hemoglobin; HDL-C, high-density lipoprotein cholesterol; LDL-C, low-density lipoprotein cholesterol; sBP, systolic blood pressure.

Table S2. Medication therapy of the T1D cohort.

	No DR<br>*N* = 181	DR<br>*N* = 128	P over-all	No DR<br>*N* = 181	Mild<br>*N* = 80	Advanced<br>*N* = 48	p.over-all	p.No DR vs Mild	p.No DR vs Advanced	p.Mild vs Advanced
Antiplatelet	45 (24.9%)	43 (33.6%)	0.122	45 (24.9%)	22 (27.5%)	21 (43.8%)	0.035	0.767	0.051	0.136
Statins	67 (37.0%)	64 (50.0%)	0.031	67 (37.0%)	37 (46.2%)	27 (56.2%)	0.041	0.307	0.075	0.361
Fibrate	0 (0.00%)	2 (1.56%)	0.171	0 (0.00%)	2 (2.50%)	0 (0.00%)	0.09	0.186	.	0.528
Insulin	181 (100%)	128 (100%)	.	181 (100%)	80 (100%)	48 (100%)	.	.	.	.

Data are shown as *n* (%) for categorical variables and median (IQR) for continuous variables.

Table S3. Medication therapy of the T2D cohort.

	No DR	DR	p.overall	No DR	Mild	Advanced	p.overall	p.No DR vs Mild	p.No DR vs Advanced	p.Mild vs Advanced
	N = 139	*N* = 125		*N* = 139	*N* = 50	*N* = 75				
Antiplatelet	40 (28.8%)	57 (45.6%)	0.007	40 (28.8%)	21 (42.0%)	36 (48.0%)	0.014	0.186	0.024	0.634
Statins	55 (39.6%)	56 (44.8%)	0.462	55 (39.6%)	23 (46.0%)	33 (44.0%)	0.674	0.944	0.944	0.971
Fibrate	8 (5.76%)	7 (5.60%)	1	8 (5.76%)	5 (10.0%)	2 (2.67%)	0.213	0.5	0.5	0.344
DM_Treatment			<0.001				.	<0.001	<0.001	0.005
NIAD	89 (64.0%)	56 (44.8%)		89 (64.0%)	28 (56.0%)	28 (37.3%)				
NIAD+Insulin	13 (9.35%)	53 (42.4%)		13 (9.35%)	20 (40.0%)	33 (44.0%)				
Insulin	4 (2.88%)	15 (12.0%)		4 (2.88%)	1 (2.00%)	14 (18.7%)				
Lifestyle	33 (23.7%)	1 (0.80%)		33 (23.7%)	1 (2.00%)	0 (0.00%)				

Data are shown as *n* (%) for categorical variables and median (IQR) for continuous variables. DM, Diabetes Mellitus; NIAD, Non-insulin antidiabetic drugs.

Table S4. Clinical characteristics according to sex and diabetes type.

Characteristics	T1D			T2D		
	**Men**	**Women**	***p*-value**	**Men**	**Women**	***p*-value**
	***N* = 146**	***N* = 163**		***N* = 138**	***N* = 126**	
Retinopathy:			1			1
No diabetic retinopathy	86 (58.9%)	95 (58.3%)		73 (52.9%)	66 (52.4%)	
Diabetic retinopathy	60 (41.1%)	68 (41.7%)		65 (47.1%)	60 (47.6%)	
Age (years)	45.6 (11.2)	46.4 (12.0)	0.583	57.0 [49.0;65.0]	60.0 [52.2;67.0]	0.087
BMI (kg/m²)	26.2 [23.4;28.9]	25.0 [22.4;28.0]	0.04	28.9 [27.0;32.5]	32.7 [29.1;37.0]	<0.001
Waist (cm)	94.1 (12.2)	85.4 (11.5)	<0.001	103 [97.8;110]	105 [98.0;114]	0.076
sBP (mmHg)	132 (16.4)	125 (19.5)	0.003	139 (17.1)	139 (20.6)	0.933
dBP (mmHg)	76.0 (10.1)	72.8 (9.82)	0.006	77.1 (10.4)	76.3 (10.6)	0.546
Hypertension (yes)	47 (32.2%)	41 (25.2%)	0.214	77 (55.8%)	72 (57.1%)	0.924
Dyslipidemia (yes)	64 (43.8%)	70 (42.9%)	0.966	62 (44.9%)	62 (49.2%)	0.567
Antiplatelet (yes)	50 (34.2%)	38 (23.3%)	0.046	48 (34.8%)	49 (38.9%)	0.573
Statins (yes)	63 (43.2%)	68 (41.7%)	0.261	51 (37.0%)	60 (47.6%)	0.103
Smoking:			<0.001			<0.001
Yes	45 (30.8%)	33 (20.2%)		37 (26.8%)	18 (14.3%)	
Former smoker	47 (32.2%)	33 (20.2%)		73 (52.9%)	14 (11.1%)	
Diabetes duration	18.0 [12.0;26.0]	23.0 [16.0;30.0]	0.002	7.00 [4.00;10.0]	10.0 [5.00;15.0]	0.003
DM Treatment						0.01
Insulin	146 (100%)	163 (100%)	0.889	9 (6.5%)	10 (7.9%)	
NIAD				89 (64.5%)	56 (44.4%)	
NIAD+ Insulin				25 (18.1%)	41 (32.5%)	
Lifestyle			0.222	15 (10.9%)	19 (15.2%)	
Glucose (mg/dL)	162 [110;215]	150 [114;206]	0.261	149 [119;176]	144 [116;186]	0.498

Creatinine (mg/dL)	0.87 [0.79;0.96]	0.67 [0.60;0.75]	<0.001	0.90 [0.79;0.97]	0.69 [0.61;0.77]	<0.001
eGFR (mL/min/1.73 m^2)	101 (13.8)	102 (14.3)	0.522	92.1 [83.5;102]	94.0 [82.5;102]	0.881
Triglycerides (mg/dL)	73.0 [56.2;92.0]	63.0 [53.0;80.5]	0.005	109 [82.0;168]	121 [90.0;166]	0.198
Total C (mg/dL)	172 [158;195]	184 [161;203]	0.088	174 [153;197]	192 [170;217]	<0.001
HDL-C (mg/dL)	57.0 [50.0;66.0]	66.5 [57.0;77.0]	<0.001	45.0 [38.0;53.0]	54.0 [45.0;61.0]	<0.001
LDL-C (mg/dL)	102 [85.3;116]	98.2 [82.0;115]	0.327	104 [83.3;122]	112 [92.4;132]	0.006
HbA1c (%)	7.40 [7.00;8.10]	7.60 [7.00;8.20]	0.298	7.40 [6.80;8.35]	7.55 [6.80;8.72]	0.495
HbA1c (mmol/mol)	57.0 [53.0;65.0]	60.0 [53.0;66.0]	0.275	57.0 [51.0;67.5]	59.0 [51.0;72.2]	0.448
Plaque:			0.396			0.051
No	93 (63.7%)	110 (67.5%)		52 (37.7%)	59 (46.8%)	
One plaque	23 (15.8%)	29 (17.8%)		34 (24.6%)	37 (29.4%)	
Multiple plaques	30 (20.5%)	24 (14.7%)		52 (37.7%)	30 (23.8%)	
FLI	27.2 [14.7;51.2]	13.0 [5.87;30.3]	0.281	68.1 [48.1;86.5]	77.7 [58.5;92.6]	0.03
Microalbuminuria (mg/L)	3.75 [2.20;6.89]	4.80 [2.35;9.40]	0.209	10.5 [5.00;19.8]	7.70 [3.55;22.1]	0.238
ACR (mg/g)	1.03 [1.02;2.10]	1.03 [1.02;4.38]	0.62	6.93 [3.72;13.5]	10.0 [4.65;21.2]	0.038

Data are shown as *n* (%) for categorical variables and median (IQR) for continuous variables. ACR, albumin-to-creatinine ratio; BMI, body mass index; C, cholesterol; sBP, diastolic blood pressure; DM, Diabetes Mellitus; eGFR, estimated glomerular filtration rate calculated according to Chronic Kidney Disease Epidemiology Collaboration (CKD-EPI) equation; FLI, fatty liver index; HbA1c, glycated hemoglobin; HDL-C, high-density lipoprotein cholesterol; IFCC, International Federation of Clinical Chemistry; LDL-C, low-density lipoprotein cholesterol; NIAD, Non-insulin antidiabetic drugs; sBP, systolic blood pressure.

Table S5. Changes in the advanced lipoprotein and glycoprotein profile according to degree of diabetic retinopathy in two independent cohorts of diabetic subjects.

Advanced Lipoprotein Profile	T1D				T2D			
	No DR	Mild	Advanced	*p*-value	No DR	Mild	Advanced	*p*-value
	n = 181	*n* = 80	*n* = 48		*N* = 139	*N* = 50	*n* = 75	
VLDL-P number								
Total (nmol/L)	30.2 (14.6)	34.9 (20.9)	34.6 (28.9)	0.123	69.1 (45.2)	83.5 (73.6)	60.3 (38.9)	0.043
Large (nmol/L)	0.81 (0.32)	0.90 (0.42)	0.88 (0.54)	0.169	1.63 (0.89)	1.91 (1.64)	1.43 (0.75)	0.041
Medium (nmol/L)	3.11 (1.79)	3.50 (2.95)	3.52 (3.81)	0.391	6.26 (6.68)	8.37 (11.7)	5.24 (3.60)	0.062
Small (nmol/L)	26.3 (12.7)	30.5 (17.8)	30.2 (24.7)	0.104	61.2 (38.6)	73.2 (61.1)	53.7 (35.2)	0.046
VLDL-P composition								
VLDL-C (mg/dL)	7.62 (5.65)	9.20 (8.04)	9.01 (9.74)	0.183	17.1 (13.4)	20.9 (18.2)	15.1 (12.1)	0.080
VLDL-TG (mg/dL)	43.0 (20.1)	49.2 (29.4)	48.9 (41.4)	0.145	98.8 (68.0)	121 (119)	85.3 (52.7)	0.039
VLDL-P size (nm)	42.2 (0.23)	42.1 (0.23)	42.1 (0.24)	0.128	42.0 (0.21)	42.0 (0.23)	42.0 (0.22)	0.446
LDL-P number								
Total (nmol/L)	1265 (193)	1285 (228)	1285 (235)	0.704	1355 (252)	1275 (277)	1277 (254)	0.045
Large (nmol/L)	182 (29.6)	182 (31.6)	182 (32.6)	0.985	174 (31.5)	174 (38.8)	172 (33.3)	0.910
Medium (nmol/L)	413 (106)	405 (122)	417 (127)	0.808	392 (129)	358 (135)	392 (138)	0.275
Small (nmol/L)	669 (95.1)	699 (119)	686 (109)	0.096	789 (124)	742 (150)	712 (121)	<0.001
LDL-P composition								
LDL-C (mg/dL)	125 (19.7)	125 (23.3)	125 (23.9)	0.990	127 (25.2)	119 (27.2)	121 (25.7)	0.082
LDL-TG (mg/dL)	15.8 (4.31)	16.3 (4.66)	16.9 (4.95)	0.285	17.4 (4.74)	17.5 (5.24)	18.1 (5.60)	0.633
LDL-P size (nm)	21.1 (0.24)	21.0 (0.25)	21.0 (0.24)	0.092	20.8 (0.23)	20.8 (0.41)	20.9 (0.25)	0.013
HDL-P number								
Total (nmol/L)	32.7 (5.95)	32.8 (7.65)	33.3 (5.89)	0.879	27.1 (5.01)	27.5 (6.74)	27.8 (5.53)	0.600
Large (nmol/L)	0.28 (0.05)	0.29 (0.06)	0.30 (0.04)	0.238	0.26 (0.04)	0.27 (0.06)	0.27 (0.05)	0.636

Medium (nmol/L)	10.8 (2.42)	10.8 (2.79)	11.3 (2.46)	0.553	8.04 (1.34)	8.08 (1.99)	8.77 (2.23)	0.013
Small (nmol/L)	21.6 (4.21)	21.7 (5.52)	21.7 (4.16)	0.992	18.8 (4.24)	19.1 (5.42)	18.8 (4.20)	0.869
HDL-P composition								
HDL-C (mg/dL)	65.6 (13.4)	65.0 (18.1)	66.4 (13.9)	0.878	49.4 (9.47)	49.6 (12.9)	52.2 (12.6)	0.200
HDL-TG (mg/dL)	13.8 (3.98)	14.6 (4.15)	15.1 (3.25)	0.083	14.0 (4.58)	14.8 (5.85)	14.2 (3.95)	0.627
HDL-P size (nm)	8.23 (0.06)	8.23 (0.06)	8.24 (0.06)	0.546	8.20 (0.07)	8.19 (0.07)	8.22 (0.08)	0.130
IDL-P composition								
IDL-C (mg/dL)	9.38 (4.43)	10.5 (4.73)	11.2 (5.53)	0.025	13.1 (4.83)	13.3 (4.71)	13.6 (5.79)	0.807
IDL-TG (mg/dL)	10.7 (3.37)	11.7 (3.68)	12.1 (4.18)	0.027	14.5 (4.13)	15.0 (3.79)	14.9 (4.69)	0.667
Other atherogenic variables								
Non-HDL-P (nmol/L)	1262 (198)	1287 (228)	1286 (244)	0.609	1397 (254)	1331 (266)	1309 (260)	0.041
Total-P/HDL-P	41.0 (10.9)	42.1 (11.2)	41.0 (11.5)	0.750	54.5 (15.1)	52.3 (16.1)	49.9 (14.1)	0.092
LDL-P/HDL-P	40.0 (10.5)	41.0 (10.8)	39.9 (10.9)	0.791	51.8 (14.3)	49.1 (15.5)	47.5 (13.1)	0.090
C total	208 (26.2)	210 (32.9)	212 (31.1)	0.601	207 (32.0)	203 (34.0)	201 (33.3)	0.489
TG total	83.3 (27.0)	91.7 (36.8)	92.9 (48.7)	0.074	142 (62.2)	157 (70.3)	132 (60.2)	0.108
Ratio VLDL	7.47 (3.87)	7.31 (3.91)	7.40 (3.72)	0.951	7.11 (3.36)	6.35 (2.22)	7.20 (3.34)	0.279
Ratio LDL	0.13 (0.02)	0.13 (0.03)	0.13 (0.03)	0.108	0.14 (0.03)	0.15 (0.04)	0.15 (0.04)	0.009
Ratio IDL	1.25 (0.31)	1.20 (0.32)	1.16 (0.24)	0.171	1.16 (0.26)	1.18 (0.23)	1.16 (0.25)	0.835
Ratio HDL	0.21 (0.06)	0.24 (0.08)	0.23 (0.07)	0.027	0.29 (0.10)	0.31 (0.13)	0.29 (0.11)	0.384
Per. VLDL	0.87 (0.03)	0.88 (0.03)	0.88 (0.03)	0.206	0.89 (0.02)	0.88 (0.03)	0.89 (0.03)	0.244
Per. LDL	0.53 (0.05)	0.55 (0.06)	0.54 (0.05)	0.091	0.59 (0.05)	0.59 (0.07)	0.56 (0.06)	0.019
Per. HDL	0.66 (0.04)	0.66 (0.05)	0.65 (0.05)	0.561	0.69 (0.04)	0.69 (0.05)	0.67 (0.06)	0.042
Advanced Glycoprotein profile								
Glyc A	5.12 (1.03)	5.16 (0.98)	5.22 (1.34)	0.839	6.77 (1.78)	7.40 (3.88)	6.53 (1.36)	0.099
Glyc B	2.02 (0.40)	2.00 (0.37)	1.97 (0.43)	0.755	2.25 (0.32)	2.40 (0.83)	2.29 (0.38)	0.178

H/W GlycA	17.1 (3.45)	16.8 (2.69)	17.0 (3.28)	0.856	21.8 (3.78)	22.4 (5.32)	21.5 (3.73)	0.427
H/W GlycB	4.88 (0.93)	4.70 (0.74)	4.85 (0.84)	0.322	5.93 (0.92)	5.97 (0.90)	5.92 (0.95)	0.942

Data are shown as mean (SD) for continuous variables. GlycA, area proportional to the concentration of the acetyl groups of N-acetylglucosamine and N-acetyl galactosamine; GlycB, area proportional to the concentration of the acetyl groups of acetyl groups of N-acetylneuraminic acid; HDL-P, high-density lipoprotein-particle; HDL-C, high-density lipoprotein-cholesterol; HDL-TG, high-density lipoprotein-triglyceride; H/W GlycA, height-to-width ratio of N-acetylglucosamine and N-acetyl galactosamine; H/W GlycB, height-to-width ratio of acetyl groups of N-acetylneuraminic acid; IDL-P, intermediate-density lipoprotein-particle; IDL-C, intermediate-density lipoprotein-cholesterol; IDL-TG, intermediate-density lipoprotein-triglyceride; LDL-P, low-density lipoprotein-particle; LDL-C, low-density lipoprotein-cholesterol; LDL-TG, low-density lipoprotein-triglyceride; per. HDL, percentage of small HDL particles; per. LDL, percentage of small LDL particles; per. VLDL, percentage of small VLDL particles; ratio HDL, HDL-C-to-HDL-TG ratio; ratio lDL, LDL-C-to-LDL-TG ratio; ratio LDL, LDL-C-to-LDL-TG ratio; ratio VLDL, VLDL-C-to-VLDL-TG ratio; VLDL-P, very low-density lipoprotein-particle; VLDL-C, very low-density lipoprotein-cholesterol; VLDL-TG, very low-density lipoprotein-triglyceride.

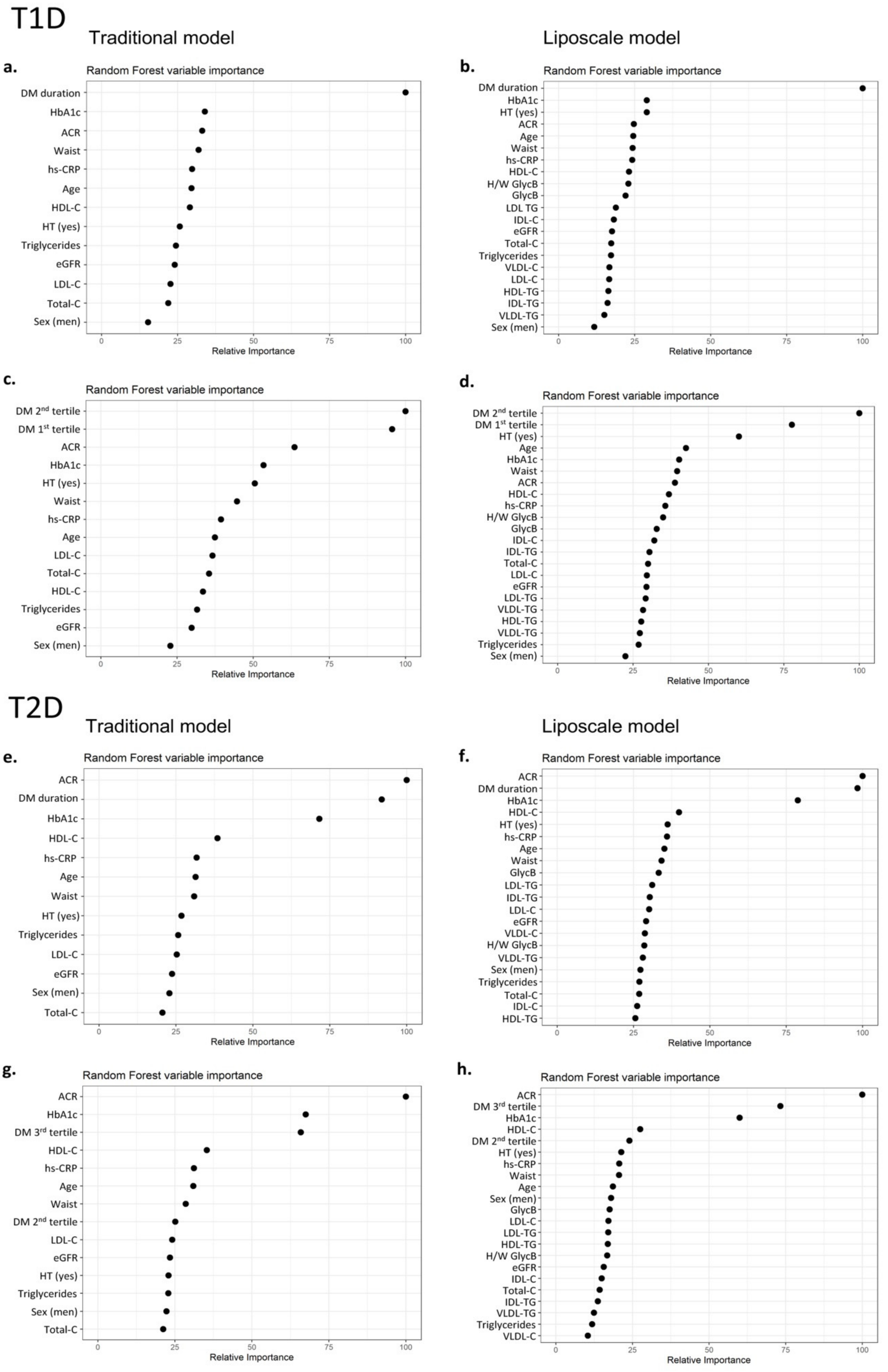
T1D
Traditional model
Liposcale model
a.
Random Forest variable importance
DM duration
HbA1c
ACR
Waist
hs-CRP
Age
HDL-C
HT (yes)
Triglycerides
eGFR
LDL-C
Total-C
Sex (men)
Relative Importance
b.
Random Forest variable importance
DM duration
HbA1c
HT (yes)
ACR
Age
Waist
hs-CRP
HDL-C
H/W GlycB
GlycB
LDL TG
IDL-C
eGFR
Total-C
Triglycerides
VLDL-C
LDL-C
HDL-TG
IDL-TG
VLDL-TG
Sex (men)
Relative Importance
c.
Random Forest variable importance
DM 2nd tertile
DM 1st tertile
ACR
HbA1c
HT (yes)
Waist
hs-CRP
Age
LDL-C
Total-C
HDL-C
Triglycerides
eGFR
Sex (men)
Relative Importance
d.
Random Forest variable importance
DM 2nd tertile
DM 1st tertile
HT (yes)
Age
HbA1c
Waist
ACR
HDL-C
hs-CRP
H/W GlycB
GlycB
IDL-C
IDL-TG
Total-C
LDL-C
eGFR
LDL-TG
VLDL-TG
HDL-TG
VLDL-TG
Triglycerides
Sex (men)
Relative Importance
T2D
Traditional model
Liposcale model
e.
Random Forest variable importance
ACR
DM duration
HbA1c
HDL-C
hs-CRP
Age
Waist
HT (yes)
Triglycerides
LDL-C
eGFR
Sex (men)
Total-C
Relative Importance
f.
Random Forest variable importance
ACR
DM duration
HbA1c
HDL-C
HT (yes)
hs-CRP
Age
Waist
GlycB
LDL-TG
IDL-TG
LDL-C
eGFR
VLDL-C
H/W GlycB
VLDL-TG
Sex (men)
Triglycerides
Total-C
IDL-C
HDL-TG
Relative Importance
g.
Random Forest variable importance
ACR
HbA1c
DM 3rd tertile
HDL-C
hs-CRP
Age
Waist
DM 2nd tertile
LDL-C
eGFR
HT (yes)
Triglycerides
Sex (men)
Total-C
Relative Importance
h.
Random Forest variable importance
ACR
DM 3rd tertile
HbA1c
HDL-C
DM 2nd tertile
HT (yes)
hs-CRP
Waist
Age
Sex (men)
GlycB
LDL-C
LDL-TG
HDL-TG
H/W GlycB
eGFR
IDL-C
Total-C
IDL-TG
VLDL-TG
Triglycerides
VLDL-C
Relative Importance
0
25
50
75
100

Figure S1. Comparison between prediction models according to diabetes duration variable, taken as continuous or as a categorized variable. In the T1D, random forest analysis for Traditional (**a**) and Liposcale (**b**) models with diabetes duration as a continuous variable, and Traditional (**c**) and Liposcale (**d**) models with diabetes duration as a categorized variable. In the T2D group, random forest analysis for Traditional (**e**) and Liposcale (**f**) models with diabetes duration as a continuous variable, and Traditional (**g**) and Liposcale (**h**) models with diabetes duration as a categorized variable. In the Random Forest plots (a, b, c, d, e, f, g, h), we show the Out Of Bag relative importance of each variable. ACR, albumin-to-creatinine ratio; BMI, body mass index; DM, diabetes mellitus; dBP, diastolic blood pressure; eGFR, estimated glomerular filtration rate calculated according to Chronic Kidney Disease Epidemiology Collaboration (CKD-EPI) equation; GlycB, area proportional to the concentration of the acetyl groups of acetyl groups of *N*-acetylneuraminic acid; HbA1c, glycated hemoglobin; HDL-C, high-density lipoprotein cholesterol; HDL-TG, high-density lipoprotein triglyceride; hs-CRP, high sensitive C reactive protein; H/W GlycB, height-to-width ratio of GlycB; IDL-C, intermediate-density lipoprotein cholesterol; IDL-TG, intermediate-density lipoprotein triglyceride; LDL-C, low-density lipoprotein cholesterol; LDL-TG, low-density lipoprotein triglyceride; VLDL-C, very low-density lipoprotein cholesterol; VLDL-TG, very low-density lipoprotein triglyceride.; sBP, systolic blood pressure Total-C, total cholesterol.

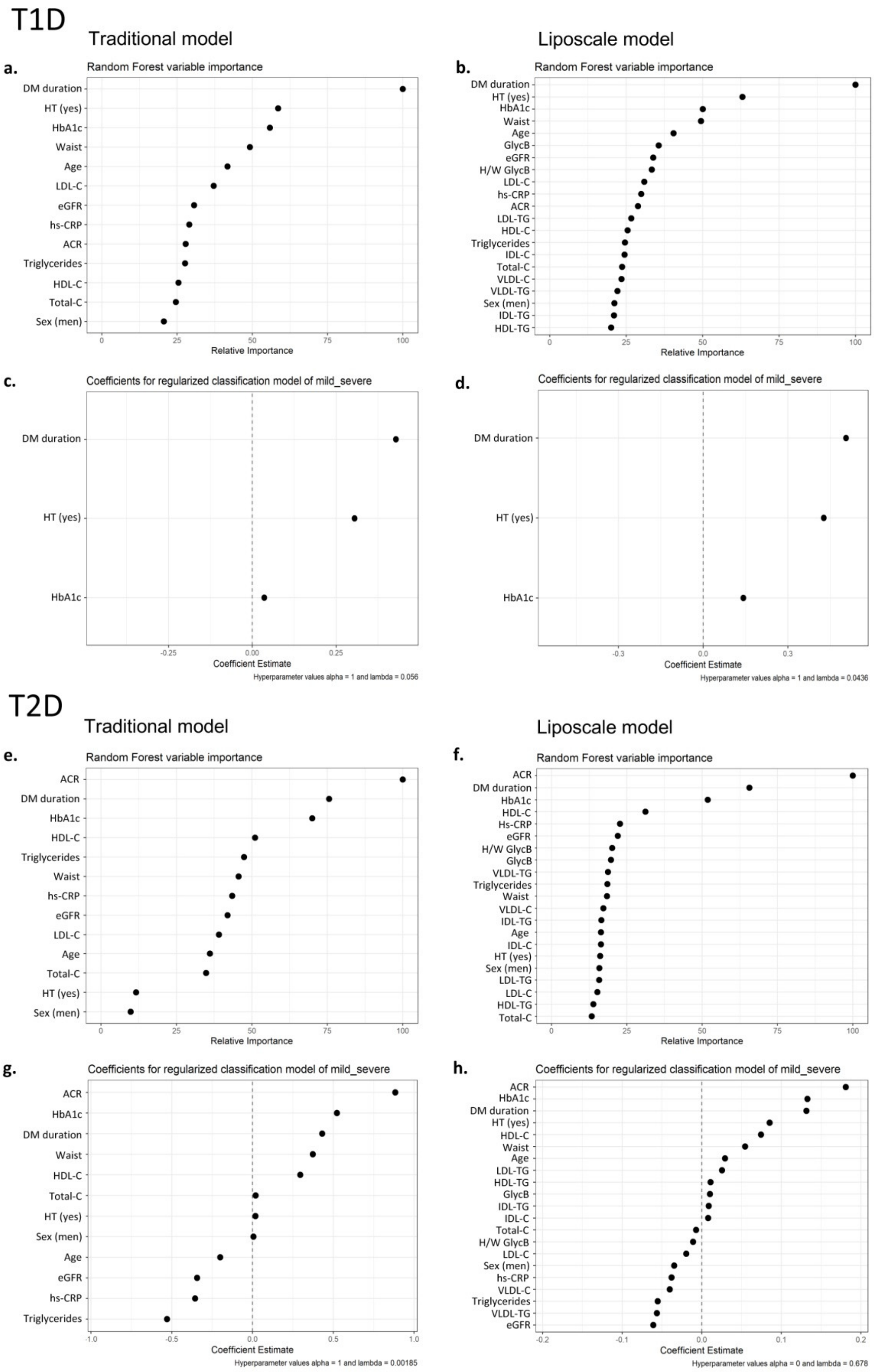

Figure S2. Traditional and Liposcale models to predict the severity of DR by assessing Mild vs Severe DR in T1D and T2D groups. In the T1D group, Random Forest analysis for traditional (**a**) and Liposcale (**b**) models, and Regression analysis for traditional (**c**) and Liposcale (**d**) models. In the

T2D group, Random Forest analysis for traditional (**e**) and Liposcale (**f**) models, and Regression analysis for traditional (**g**) and Liposcale (**h**) models. In the Random Forest plots (a, b, e, f), we show the Out Of Bag relative importance of each variable. In the regularized classification models (c, d, g, h) we show the value of the coefficients in the logistic regression after variable normalization. ACR, albumin-to-creatinine ratio; DM, diabetes mellitus; eGFR, estimated glomerular filtration rate calculated according to Chronic Kidney Disease Epidemiology Collaboration (CKD-EPI) equation; GlycB, area proportional to the concentration of the acetyl groups of acetyl groups of N-acetylneuraminic acid; HbA1c, glycated hemoglobin; HDL-C, high-density lipoprotein-cholesterol; HDL-TG, high-density lipoprotein-triglyceride; hs-CRP, high sensitive C reactive protein; H/W GlycB, height-to-width ratio of GlycB; HT, hypertension; IDL-C, intermediate-density lipoprotein-cholesterol; IDL-TG, intermediate-density lipoprotein-triglyceride; LDL-C, low-density lipoprotein-cholesterol; LDL-TG, low-density lipoprotein-triglyceride; VLDL-C, very low-density lipoprotein-cholesterol; VLDL-TG, very low-density lipoprotein-triglyceride; Total-C, total cholesterol.

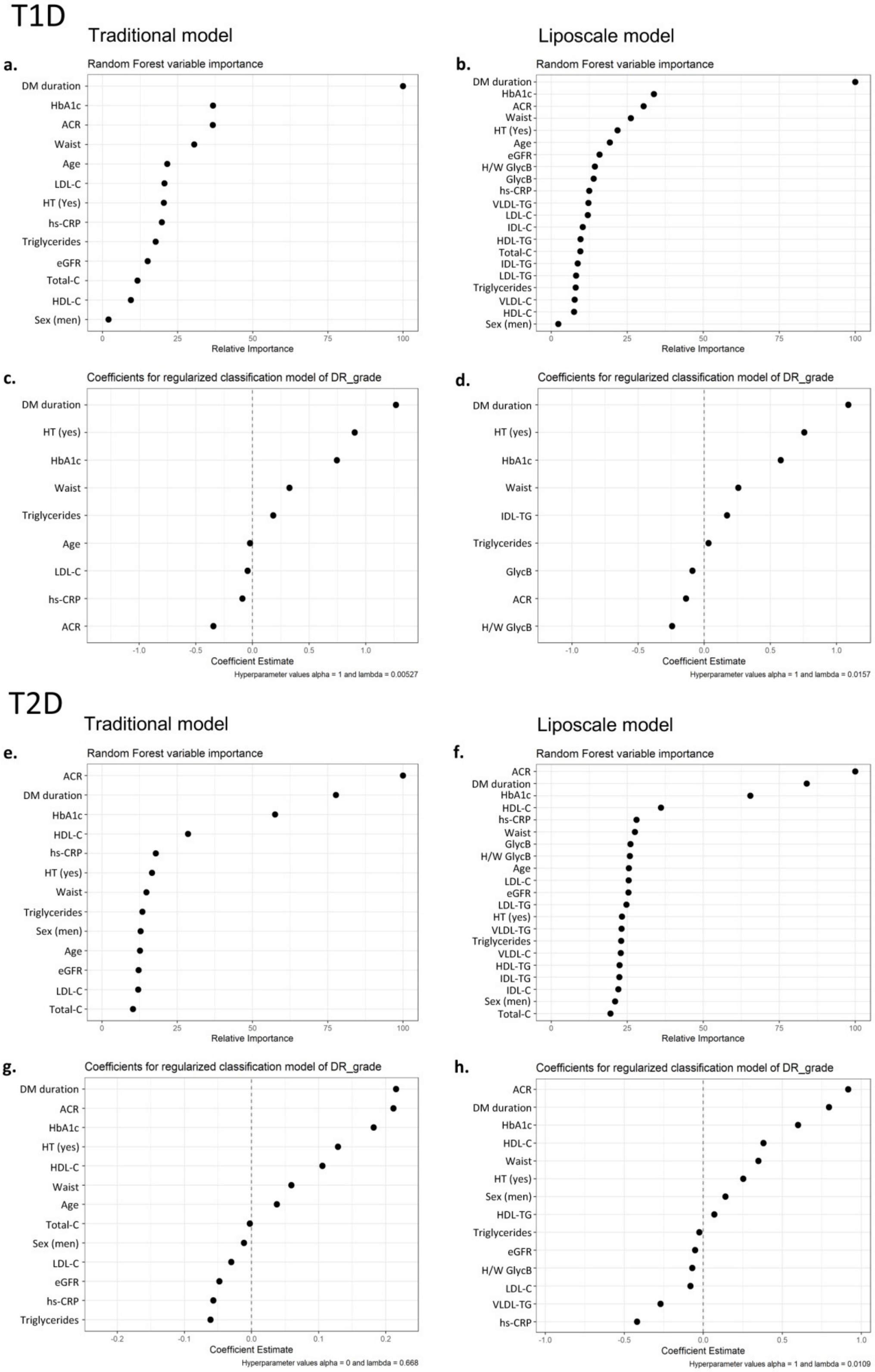

Figure S3. Traditional and Liposcale models to predict the severity by assessing Not vs Severe DR in T1D and T2D groups. In the T1D group, Random Forest analysis for traditional (**a**) and Liposcale

(**b**) models, and Regression analysis for traditional (**c**) and Liposcale (**d**) models. In the T2D group, Random Forest analysis for traditional (**e**) and Liposcale (**f**) models, and Regression analysis for traditional (**g**) and Liposcale (**h**) models. In the Random Forest plots (a, b, e, f), we show the Out Of Bag relative importance of each variable. In the regularized classification models (c, d, g, h) we show the value of the coefficients in the logistic regression after variable normalization. ACR, albumin-to-creatinine ratio; DM, diabetes mellitus; eGFR, estimated glomerular filtration rate calculated according to Chronic Kidney Disease Epidemiology Collaboration (CKD-EPI) equation; GlycB, area proportional to the concentration of the acetyl groups of acetyl groups of N-acetylneuraminic acid; HbA1c, glycated hemoglobin; HDL-C, high-density lipoprotein-cholesterol; HDL-TG, high-density lipoprotein-triglyceride; hs-CRP, high sensitive C reactive protein; H/W GlycB, height-to-width ratio of GlycB; HT, hypertension; IDL-C, intermediate-density lipoprotein-cholesterol; IDL-TG, intermediate-density lipoprotein-triglyceride; LDL-C, low-density lipoprotein-cholesterol; LDL-TG, low-density lipoprotein-triglyceride; VLDL-C, very low-density lipoprotein-cholesterol; VLDL-TG, very low-density lipoprotein-triglyceride; Total-C, total cholesterol.

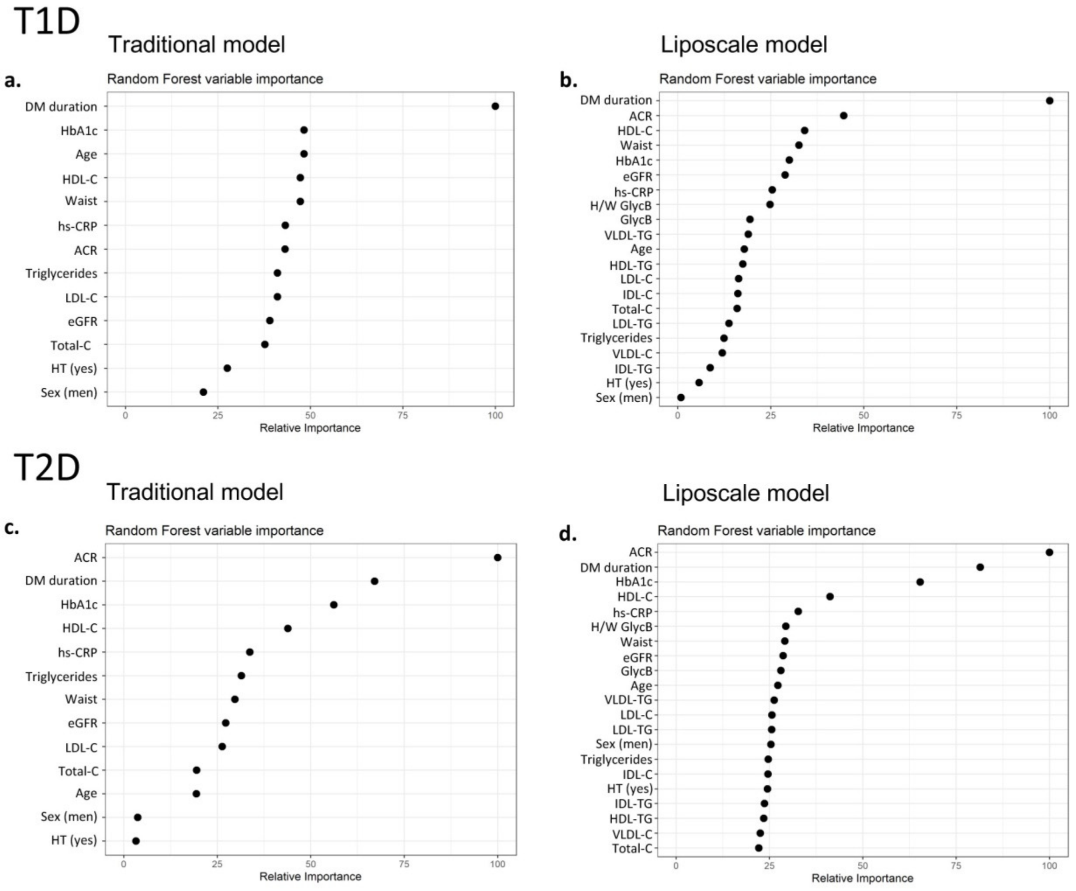

Figure S4. Traditional and Liposcale models to predict the severity by assessing DR stages in T1D and T2D groups. In the T1D group, Random Forest analysis for traditional (**a**) and Liposcale (**b**) models. In the T2D group, Random Forest analysis for traditional (**c**) and Liposcale (**d**) models. In the Random Forest plots (a, b, c, d), we show the Out Of Bag relative importance of each variable. ACR, albumin-to-creatinine ratio; DM, diabetes mellitus; eGFR, estimated glomerular filtration rate calculated according to Chronic Kidney Disease Epidemiology Collaboration (CKD-EPI) equation; GlycB, area proportional to the concentration of the acetyl groups of acetyl groups of N-acetylneuraminic acid; HbA1c, glycated hemoglobin; HDL-C, high-density lipoprotein-cholesterol; HDL-TG, high-density lipoprotein-triglyceride; hs-CRP, high sensitive C reactive protein; H/W GlycB, height-to-width ratio of GlycB; HT, hypertension; IDL-C, intermediate-density lipoprotein-cholesterol; IDL-TG, intermediate-density lipoprotein-triglyceride; LDL-C, low-density lipoprotein-cholesterol; LDL-TG, low-density lipoprotein-triglyceride; VLDL-C, very low-density lipoprotein-cholesterol; VLDL-TG, very low-density lipoprotein-triglyceride; Total-C, total cholesterol.
